# Trust Attacks and Defense in the Social Internet of Things: Taxonomy and Simulation-Based Evaluation

**DOI:** 10.3390/s25247513

**Published:** 2025-12-10

**Authors:** Chunying Zhang, Siwu Lan, Liya Wang, Lu Liu, Jing Ren

**Affiliations:** 1 College of Science, North China University of Science and Technology, Tangshan 063210, China; zchunying@ncst.edu.cn (C.Z.); lansw@stu.ncst.edu.cn (S.L.); liulu_hblg@ncst.edu.cn (L.L.); renjing@ncst.edu.cn (J.R.); 2 Hebei Key Laboratory of Data Science and Application, North China University of Science and Technology, Tangshan 063210, China; 3 The Key Laboratory of Engineering Computing in Tangshan City, North China University of Science and Technology, Tangshan 063210, China; 4 Hebei Engineering Research Center for the Intelligentization of Iron Ore Optimization and Ironmaking Raw Materials Preparation Processes, North China University of Science and Technology, Tangshan 063210, China

**Keywords:** social internet of things, trust management, trust attacks, simulation comparison

## Abstract

The Social Internet of Things (SIoT) combines social networks and the Internet of Things, enabling closer interaction between devices, users, and services. However, this interaction brings risks of trust attacks. These trust attacks not only affect the stability of SIoT systems but also threaten personal privacy and data security. This paper provides a decade-long review of SIoT trust attack research. First, it outlines the SIoT architecture, social relationship types, concept of trust, and trust management processes. It maps seven attacks—bad mouthing attack (BMA), ballot stuffing attack (BSA), self-promoting attack (SPA), discriminatory attack (DA), whitewashing attack (WWA), on-off attack (OOA), and opportunistic service attack (OSA)—clarifying their mechanisms and traits. Next, we synthesize the literature on SIoT trust models, enumerate which attack types they address, and classify defense strategies. It then conducts simulation-based comparative experiments on trust attacks to reveal their impact on node trust and transaction processing, compares attack capabilities along disruption speed, attack strength, and stealthiness, and summarizes attack surfaces with corresponding defense recommendations to better guide the design of SIoT trust management schemes. Finally, we identify open challenges and future research directions, to support the development of new trust management models better equipped to address evolving trust attacks.

## 1. Introduction

The Social Internet of Things (SIoT), as an architectural paradigm that integrates social networking concepts into the Internet of Things (IoT) [1], is increasingly becoming a critical infrastructure supporting large-scale heterogeneous terminal collaboration and service innovation in smart cities, vehicle-to-everything (V2X) networks, and industrial internet. Unlike traditional IoT, which focuses solely on physical interconnectivity between devices, SIoT centers on constructing and leveraging social relationships among “people-to-people”, “people-to-devices”, and “devices-to-devices” to endow smart devices with social attributes [2]. This paradigm enables personalized, context-aware, and cross-domain collaborative services by leveraging social networks, interaction histories, and contextual information. For example, smart home systems can adjust lighting and background music based on users’ routines and emotional states. In smart cities, systems can optimize route planning and recommend personalized public services using user interaction data from public facilities.

In the open and dynamic SIoT ecosystem, reliable interactions among entities fundamentally rely on trust. Trust management is therefore a core mechanism for ensuring service quality and system security [3]. By evaluating nodes’ historical behavior, interaction feedback, and contextual information, an effective trust management mechanism helps locate reliable services in a large, dynamic entity space and filter out untrustworthy nodes. Trust management therefore underpins service performance and user experience and is essential for the reliable operation of collaborative processes in open SIoT environments.

However, precisely because trust management occupies a pivotal position in the SIoT functional framework, it naturally becomes a primary target of malicious attacks and a major security challenge for SIoT [4]. Unlike traditional security frameworks that rely mainly on static identity authentication and cryptographic mechanisms, SIoT trust evaluation focuses on dynamically and continuously characterizing node behavioral reliability and interaction quality, which makes it particularly vulnerable to manipulation [5]. Trust attacks typically involve attackers employing strategies such as fabricating evaluation information (e.g., self-promoting attack, SPA), colluding to manipulate reputation (e.g., bad mouthing attack, BMA; ballot stuffing attack, BSA), selectively providing services (e.g., discriminatory attack, DA), or executing dynamic behaviors (e.g., on-off attack, OOA; whitewashing attack, WWA; opportunistic service attack, OSA). These strategies interfere with trust computation to inflate the trust of attackers or their colluders, or to defame honest nodes. Such attacks not only directly undermine the accuracy of trust-based decision-making—such as service discovery and resource allocation—but also potentially erode the structure and evolution of entire social networks, ultimately leading to the collapse of the trust system. Therefore, this paper aims to systematically review and classify trust attacks in SIoT and the corresponding defense strategies adopted in existing trust management models. In addition, we conduct simulation experiments to analyze the behavioral characteristics and system-level impacts of representative trust attacks, providing evidence to support the design of more robust and attack-resilient SIoT trust frameworks.

### 1.1. Existing Review Studies on SIoT

Regarding the knowledge base and current research on SIoT, existing review articles have already outlined the field from multiple perspectives. To position our contribution and enable a more focused critique, we first summarize representative surveys published between 2015 and 2024 [6,7,8,9,10,11,12,13,14] and statistically analyze their thematic coverage, as shown in Table 1. The results indicate that current research hotspots mainly concentrate on SIoT architectures, fundamental trust concepts and attributes, trust management processes and components, classifications of trust models, and trust attacks in SIoT. By contrast, topics such as applications, platforms, tools, and datasets are discussed only sporadically and lack systematic treatment.

However, while various reviews cover numerous topics, their emphases differ. Guo et al. [6], Abdelghani et al. [7], and Chahal et al. [9] focus on detailing trust management processes and their components within SIoT, while comparing and analyzing existing trust management models. Roopa et al. [8] emphasizes the foundational theories and key domains of SIoT (service discovery and composition, network navigability and relationship management). Khan et al. [10] centers its research on the latest advancements in trust management. Rad et al. [11] provides a comprehensive overview of core components in SIoT, including architecture, relationship management, trust management, web services, information processing, and tool support. Alam et al. [12] and Sagar et al. [14] emphasize the theoretical foundations of trust management in SIoT, discussing existing trust management research from multiple perspectives. Bangui et al. [13] introduces existing work across various SIoT application domains in different environments.

A synthesis of these reviews shows that, although most of them discuss “trust attack threats in SIoT” and enumerate typical attack types (e.g., BMA, BSA, SPA, OOA, OSA), these attacks are usually treated only as one element within broader trust management discussions. The treatment largely remains at the level of listing attack categories, and there is still a lack of surveys that take “trust attacks” as the central theme. From this perspective, this paper concentrates on a systematic analysis of seven common SIoT trust attacks, providing a more comprehensive and in-depth examination of trust-attack issues, building upon existing review work.

### 1.2. Comparison with Existing Reviews

Through statistical analysis of topics in existing review literature, this paper establishes four high-frequency themes—SIoT architecture, social relationships, trust concepts and attributes, and trust management processes—as foundational background content. Building upon this groundwork, the research focus shifts to trust attacks within SIoT, leading to further analysis. The included content and comparisons with other literature are shown in Table 2.

Table 2 further shows that most existing surveys provide only a brief introduction to trust attacks, devote limited attention to defense strategies, and almost never include simulation studies of attack mechanisms. In contrast to prior work that mainly adopts an architectural or generic trust-management view, this paper takes trust attacks as its primary lens and builds a systematic review framework around attack types, underlying mechanisms, defense strategies, and simulation-based evaluation, thereby addressing the current lack of attack-centric research in this field.

### 1.3. Main Contributions and Article Structure of This Paper

The main contributions of this paper include:

- A comprehensive introduction to SIoT architecture, social relationships, trust concepts, attributes, and a detailed trust management flowchart is provided and analyzed.
- Seven trust attacks—BMA, BSA, DA, OOA, WWA, OSA, and SPA—are introduced with analysis of their characteristics and mechanisms through schematic visualizations.
- Comprehensively interpret the relevant literature proposed in the past 10 years that can cope with trust attacks, summarize the types of trust attacks that can be coped with by the relevant trust models, and classify the defense strategies for the corresponding trust attacks.
- Construct a virtual node interaction scenario and design trust-attack simulations with varying attacker ratios to assess their impact on system security. The experiments reveal their effects on node trust and transaction processing, comparatively analyze attack capabilities along disruption speed, attack strength, and stealthiness, and summarize the attack surfaces and recommended defense strategies for each trust attack to better guide SIoT trust-management design.
- For the current research trend of trust management in SIoT, future research directions and technical challenges are further proposed.

The remainder of this paper is organized as follows. Section 2 introduces the general architecture and types of social relationships within the SIoT. Section 3 outlines the concept of trust and the trust management process within trust management. Section 4 reviews trust models in the social Internet of Things over the past decade. Section 5 presents a comparative simulation study of trust attacks. Section 6 discusses challenges and future research. Section 7 concludes the paper.

## 2. Overview of SIoT

### 2.1. Architecture of SIoT

**Figure 1:**
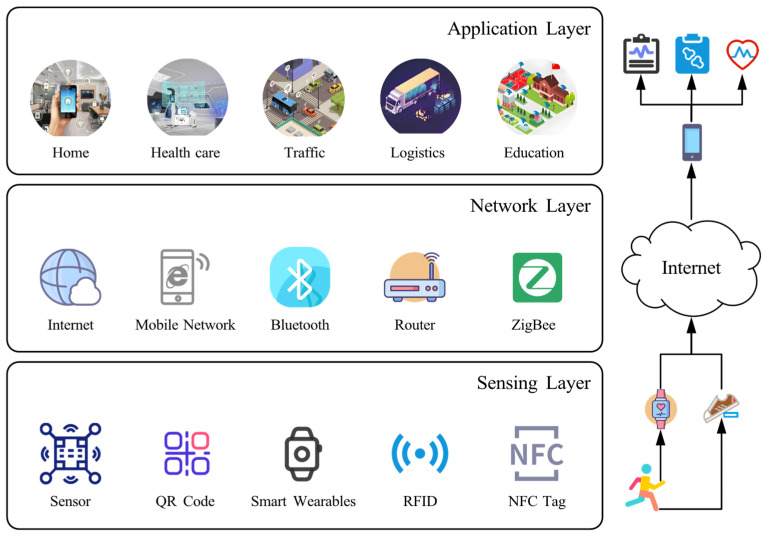
The general architecture of SIoT.

SIoT architectures define the structural framework that supports social interactions among devices, and most existing work adopts a layered design, typically with three layers. Qasabeh et al. [15] and Shahab et al. [16] propose a three-layer architecture comprising a base/object layer, an intermediate/composite layer, and an application layer, where the base layer manages data and ontology, the intermediate layer handles object interactions, and the application layer supports user–application interaction. Chahal et al. [9], Alam et al. [12], and Bouazza et al. [17] instead describe a three-layer model with perception, network, and application layers, responsible respectively for data collection, data transmission, and service provision. Building on [9], Kumari et al. [18] extend this structure to four layers—sensing, network, application, and social—where the social layer enables social interactions among devices. A generic layered SIoT architecture summarizing these designs is illustrated in Figure 1.

In the general architecture shown in Figure 1, the perception layer at the bottom collects environmental data from sensors, QR codes, smart wearables, and other devices [19]. The network layer in the middle relies on communication technologies such as Wi-Fi, Bluetooth, and Zigbee to transmit data reliably. The application layer at the top provides user-facing services and applications, including smart home [20], smart healthcare [21], smart transportation [22], smart logistics [23], and smart campus [24]. Through this layered design, SIoT supports the end-to-end process from data collection to transmission and service delivery across multiple domains. For example, in a sports health monitoring scenario, the perception layer comprises smart bracelets and smart insoles that continuously measure runners’ heart rate, gait, and other physiological and activity data. The network layer forwards this data to cloud servers via smartphones and the Internet, while the application layer offers advanced health monitoring and social interaction functions through cloud-based applications.

### 2.2. SIoT Relationship Types

**Figure 2:**
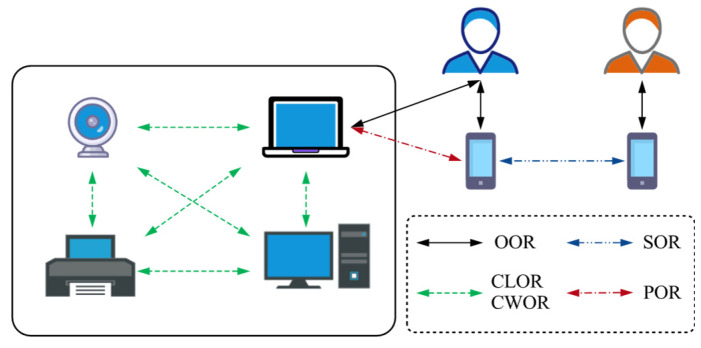
Types of relationships in SIoT.

In SIoT systems, diverse social relationships can be constructed between objects based on interaction behaviors, rule-based policies set by the owner, and communication connections [25]. These relationships can be categorized into two types according to the type of interacting objects: user-object (UO) relationships and object-object (OO) relationships. When developing SIoT applications, these relationship types must be carefully considered, as they are crucial to application success and strongly dependent on the application domain. Figure 2 illustrates five typical social relationship types.

- Ownership Object Relationship (OOR): relationship between objects owned by the same user. The user can jointly manage these objects, enabling cross-device collaboration and enhancing interconnectivity among them.
- Social Object Relationship (SOR): relationship between objects owned by different users, formed through social connections. It supports information sharing and social interaction, thereby expanding the social network.
- Parental Object Relationship (POR): relationship between homogeneous objects produced by the same batch or manufacturer. This stable association facilitates mutual identification and coordination among objects and improves system management efficiency.
- Co-Location Object Relationship (CLOR): relationship between objects that are colocated in the same geographic area. It supports location-aware services and localized collaboration, helping to optimize resource allocation.
- Co-Work Object Relationship (CWOR): relationship between objects that work together to achieve common goals. It emphasizes task-oriented collaboration and enhances the overall effectiveness of the system.

In practical applications, different types of social relationships are assigned different weights according to their importance in a given context, which influences decision-making and helps optimize overall system performance [26].

## 3. Overview of Trust Management

### 3.1. Concepts and Attributes of Trust

The concept of trust has been defined differently across disciplines such as sociology, psychology, communication, economics, management, and computer science, reflecting its abstract and complex nature. In sociology, trust is often seen as an individual’s willingness to accept vulnerability or take risks in interactions with others [27]. In psychology, it is viewed as a mental attitude based on subjective beliefs about others’ reliability and honesty, and the expectation that they will not intentionally cause harm [28]. In communication studies, trust involves reliance on information sources, media, and communication agencies for the accuracy, completeness, and reliability of information, and is a key element of effective communication and audience understanding [29]. In economics, trust is understood as the voluntary act of placing resources at another’s disposal, driven by expectations about others’ future behavior and associated benefits [30]. In management, it describes the extent to which a trusting party is willing to accept potential negative consequences when relying on someone or something [31]. In computer science, trust is the degree to which a system, component, user, or service is perceived to be reliable, secure, and honest when performing operations [32].

Overall, trust is a complex socio-cognitive phenomenon, but in a general sense it can be defined as follows: when entity A assumes that entity B will behave in line with A’s expectations, A is said to trust B [33]. This definition implies that trust involves assumptions, expectations, and observable behavior, and is inherently linked to risk and uncertainty, which makes it difficult to predict or quantify in a fully automated way. In addition, trust exhibits several key attributes that further characterize its nature and behavior; some common attributes are summarized below.

- Directness: Trust can be based on the experience of direct interaction between the granting party (subject) and the trusted party (object).
- Indirectness: When there’s no direct interaction between parties, trust decisions often rely on third-party recommendation information.
- Subjectivity: Trust is a subjective judgment made by the trustor, and different entities may apply different criteria when assessing trustworthiness, even under the same context and observable behaviors.
- Objectivity: Trust can be calculated by considering specific attributes of the trusted party.
- Localization: Trust is defined with respect to a specific trustor–trustee pair, and trust values may differ across different pairs even for the same entity.
- Global: Global trust, also known as reputation, is usually accumulated and propagated through the interaction of multiple nodes.
- Asymmetry: Trust asymmetry means entity A trusting B doesn’t imply B trusts A, and the degree of A’s trust in B may differ from B’s in A.
- Contextual relevance: The trust between the grantor and the trusted party will change with the change of contextual information.
- Decay: Trust tends to decay over time. If entity A does not interact with entity B for a period of time, then A’s perception of B decreases, leading to a decrease in the level of trust.

In SIoT, smart devices and users are connected through social relationships, and mutual trust must be established to support reliable information exchange, resource sharing, and collaboration. Trust therefore plays a critical role in SIoT and directly influences the security, stability, and overall efficiency of the system.

### 3.2. Processes for Trust Management

**Figure 3:**
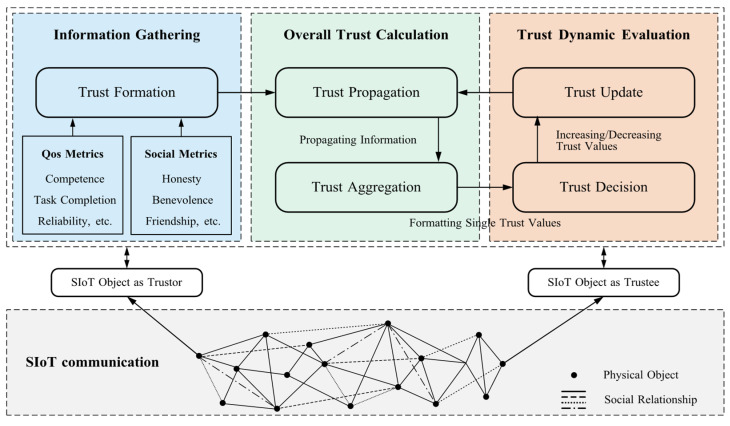
The general process of trust management.

Trust management is the process of establishing and maintaining trustworthy relationships between entities, which can be conceptualized in two forms: the process of making one entity trustworthy to other entities, and the process of assessing the trustworthiness of other entities from the perspective of a particular entity [34]. In SIoT environments, it serves as an intermediate layer between service requesters and providers, enabling trusted interactions for various security services [35]. With the rapid development of SIoT, it has become a critical research issue [36]. As illustrated in Figure 3, a typical trust-management process consists of five main steps: trust composition, trust propagation, trust aggregation, trust decision-making, and trust update.

1. Trust composition: Trust composition is the foundation of the trust management process. According to the type of social relationship, trust components are mainly divided into two categories: user-device trust (Quality of Service, QoS), which reflects competence, task completion, and reliability; and user-user trust (social trust), which captures factors such as honesty, benevolence, and friendship.
2. Trust propagation: Trust propagation is the process by which trust information spreads from one node to other nodes in the network. In this process, nodes influence the trust assessment of other nodes by passing trust values to each other or through the accumulation of direct experience. Usually, trust propagation can be categorized into distributed, central, and hybrid.
  - Distributed [37]: Each node independently collects experience, updates its own trust assessment, and exchanges trust values with others. This yields decentralized, flexible, and fault-tolerant propagation without a central controller.
  - Centralized [38]: A central node collects, stores, and disseminates trust information. Other nodes query this node to update their trust assessments, which introduces a potential single point of failure.
  - Hybrid [39]: Combines distributed and centralized mechanisms to balance scalability, robustness, and management overhead.
3. Trust aggregation: In practice, trust assessments from a single source rarely capture the full trustworthiness of a node, so it is necessary to aggregate information from multiple trust sources and factors to obtain more accurate results. Existing trust aggregation techniques include weighted sum [40,41], belief theory [42,43], Bayesian theory [44,45], fuzzy logic [46,47], regression analysis [48,49], blockchain [50,51], and machine learning [52,53,54].
4. Trust decision making: Trust decision-making evaluates the aggregated trust value to determine whether another entity is trustworthy and to choose the corresponding action. Existing approaches are typically categorized into policy-based decisions and threshold-based decisions.
5. Trust update: A trust management system must dynamically update trust values over time or when new interactions occur, so that recent behavioral changes are reflected promptly. This process, known as trust update, is typically categorized into time-driven, event-driven, and hybrid update mechanisms.
  - Time-driven [55]: Trust values are updated automatically at fixed time intervals, independent of specific external events.
  - Event-driven [56]: Trust values are updated when certain events occur, such as node interactions, user feedback, or detected abnormal behaviors.
  - Hybrid-driven [57]: Combines time-driven and event-driven mechanisms to update trust values.

## 4. Trust Attacks and Their Defense Strategies

### 4.1. Overview of Trust Attacks

A trust attack involves an attacker exploiting existing trust relationships between systems, networks, devices, or users. By manipulating, forging, or tampering with trust-related information, the attacker disrupts the normal trust mechanism for malicious purposes [58]. In network security classification, trust attacks differ from common ones like malware, phishing, and DoS (Denial of Service) attacks in aspects such as targets, modes, covertness, influence scope, and prevention difficulty, as summarized in Table 3.

### 4.2. Common Trust Attacks

In SIoT, nodes obtain services through social interactions, but a sufficient level of trust must first be established. Some malicious nodes may use interactions to launch trust attacks to gain their benefits at the expense of other nodes, thus disrupting system functionality. There are many different types of trust attacks, and seven common ones are as follows.

#### 4.2.1. Bad-Mouthing Attack (BMA)

**Figure 4:**
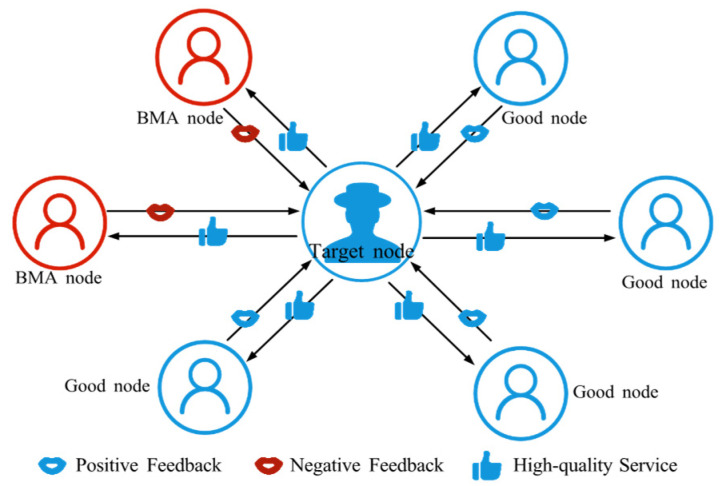
The schematic diagram of BMA. BMA nodes provide negative feedback to nodes delivering high-quality services.

BMA refers to an attacker’s efforts to reduce the trust of a target node by posting a large number of false negative evaluations to it [59], as shown in Figure 4. These evaluations may falsely accuse the node’s equipment, service quality, or interaction behavior, making the node seem untrustworthy to others. Consequently, the target node’s reputation is damaged, affecting its normal operation and potentially excluding it from resource sharing and collaboration.

#### 4.2.2. Ballot Stuffing Attack (BSA)

**Figure 5:**
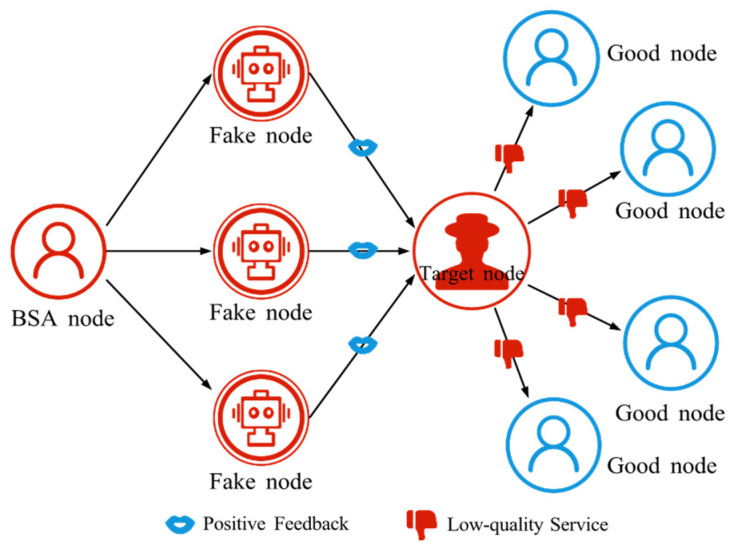
The schematic diagram of BSA. BSA nodes manipulate fake nodes to provide positive feedback to target nodes, thereby enhancing trust in the target nodes.

BSA is a malicious attack to influence the final result by manipulating the voting or scoring system [60], as shown in Figure 5. The attacker controls bots or multiple fake identities to cast a large number of votes in a short period, thereby altering the system’s evaluation of a node, service, or user. As a result, the system may incorrectly boost the reputation of attacking nodes and downgrade honest nodes, leading to distorted trust assessments.

#### 4.2.3. Self-Promoting Attack (SPA)

**Figure 6:**
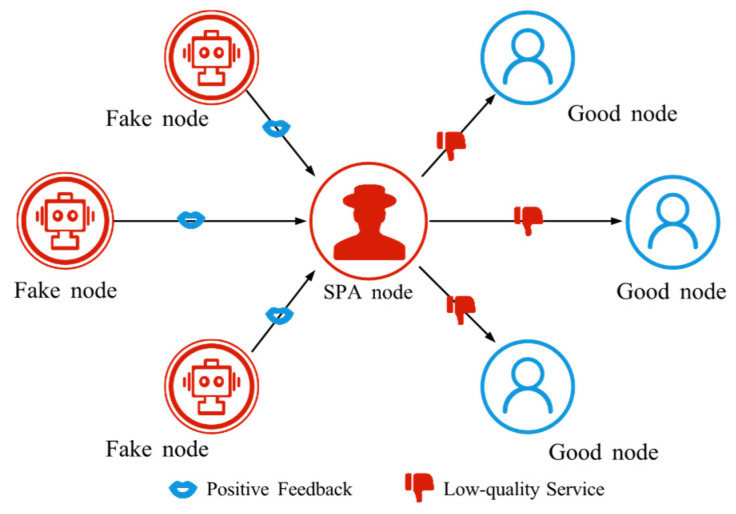
The schematic diagram of SPA. SPA nodes manipulate fake nodes to provide positive feedback, thereby enhancing their own trust.

SPA involves an attacker rapidly boosting their trust value by frequently posting positive self-comments, as shown in Figure 6. This behavior misleads the trust system into treating the attacker as highly reputable, even though its actual behavior is untrustworthy. The attacker then exploits this high trust to conduct malicious activities and disrupt the normal order of SIoT.

#### 4.2.4. Discriminatory Attack (DA)

**Figure 7:**
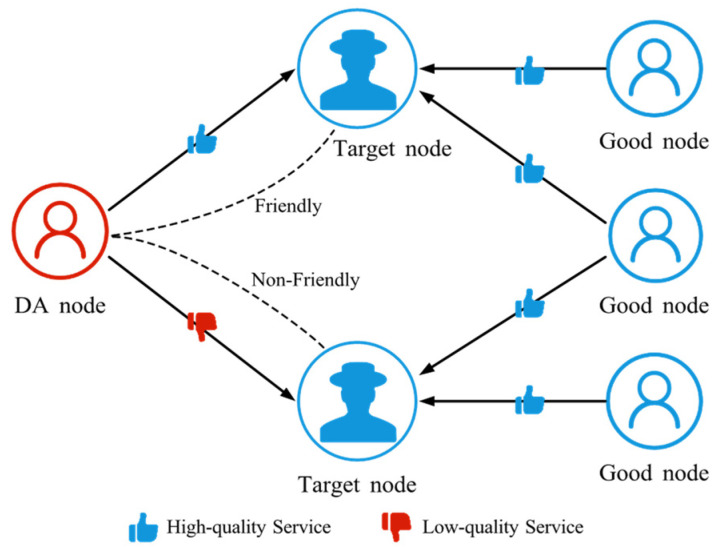
The schematic diagram of DA. DA nodes provide high-quality service to friendly nodes and low-quality service to non-friendly nodes.

DA refers to an attacker selectively manipulating trust relationships by providing high-quality services to some nodes and low-quality services to others, as shown in Figure 7. This creates imbalances in perceived service quality: certain nodes gain higher reputations in the trust management system, while others experience reduced trust. Over time, such biased service behavior and uneven trust scores distort system data, produce misleading trust assessments, and ultimately degrade the efficiency of resource allocation and collaboration across the network.

#### 4.2.5. Whitewashing Attack (WWA)

**Figure 8:**
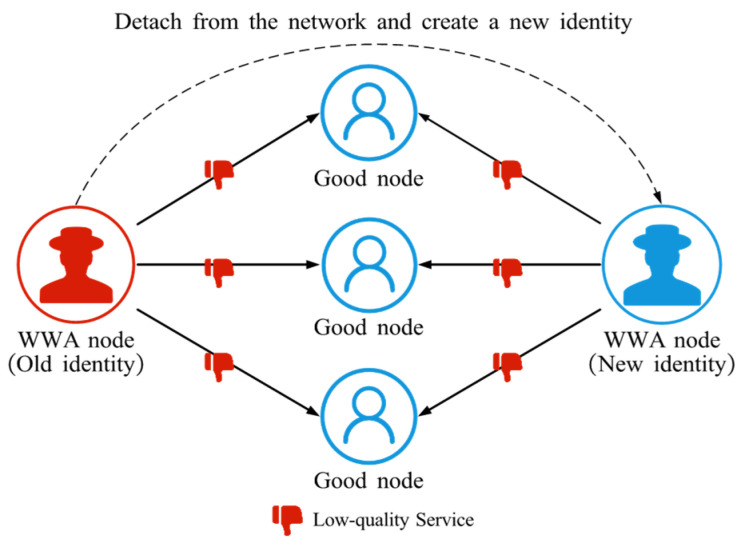
The schematic diagram of WWA. WWA nodes detach from the network when their own reputation is low and rejoin the network under a new identity to engage in malicious activities.

WWA refers to the behavior of an attacker who, after being detected, leaves the network and re-enters with a new identity to escape the penalties associated with its previous behavior [61], as illustrated in Figure 8. By repeatedly resetting its identity, the attacker effectively bypasses the trust management mechanism and can continue to damage other nodes and disrupt normal network operation.

#### 4.2.6. On/Off Attack (OOA)

**Figure 9:**
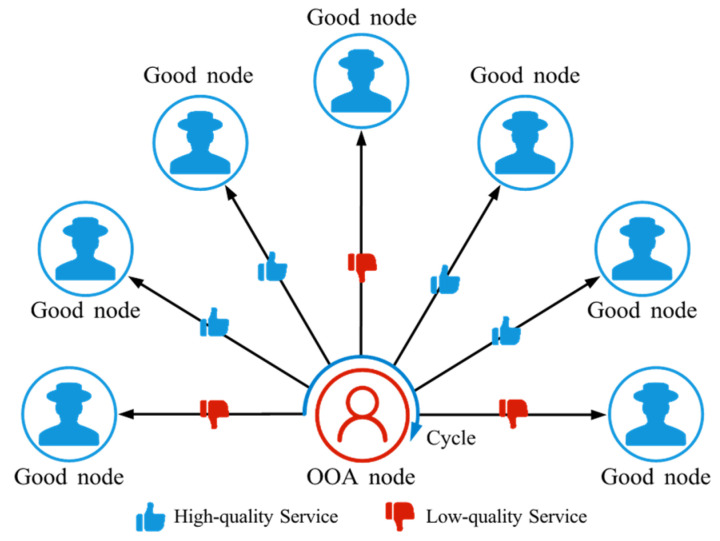
The schematic diagram of OOA. OOA nodes periodically alternate between providing high-quality services and low-quality services.

OOA is a strategic attack pattern in which an attacker alternates between providing high-quality and low-quality services to confuse the trust management system and evade detection [62], as shown in Figure 9. This unstable behavior makes it difficult for the system to identify their true intentions, leading to the fact that they can be active in the network for a long period of time and intermittently commit damage.

#### 4.2.7. Opportunistic Service Attack (OSA)

**Figure 10:**
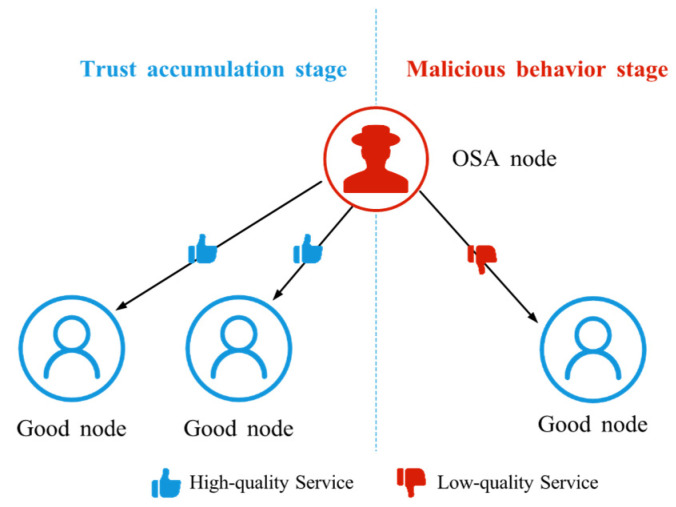
The schematic diagram of OSA. OSA nodes provide high-quality services during the trust accumulation stage, then switch to low-quality services once a certain threshold is reached.

OSA is a strategy in which an attacker initially provides high-quality services when its reputation is low in order to quickly accumulate trust; once its reputation reaches a high level, it begins to behave maliciously, as shown in Figure 10. By exploiting the protection afforded by a high trust score, the attacker can conceal its true intent and maximize the damage it causes, seriously undermining system stability and security. OSA is often regarded as one of the most sophisticated trust attacks, because the attacker must understand the operation of the trust model and be able to anticipate how its behavior will affect the evolution of trust values.

### 4.3. Trust Models in Existing Research and Their Classification

This review covers SIoT trust management studies published between 2016 and 2025. Relevant works were retrieved from Google Scholar using (“SIoT” OR “Social Internet of Things” OR “Social IoT”) AND (“trust” OR “trust model” OR “trust attack” OR “trust management”), and screened by abstract to include only studies that explicitly address SIoT scenarios and analyze trust management models involving trust attacks.

#### 4.3.1. Based on Machine Learning

Machine learning-based (ML-based) trust models can synthesize multiple trust features, handle complex nonlinear relationships, predict future trust behaviors from historical data, and have attracted increasing attention from scholars in recent years.

As summarized in Table 4, multilayer perceptrons (MLP) are the most widely used machine-learning models for trust assessment. Their popularity mainly comes from their flexible architecture, which can learn complex nonlinear dependencies among trust features. Other models also provide effective options for building trust assessments. For example, incremental support vector machine (iSVM) enables fast updates under streaming or evolving interaction data, while long short-term memory (LSTM) networks capture the temporal dynamics of entity behavior. Together, these approaches improve both the accuracy and adaptability of SIoT trust models.

Despite these advances, ML-based trust models still exhibit limitations. They typically require substantial labeled training data, which are costly or impractical to obtain in SIoT environments, and the high computational cost of training complex models makes them less suitable for latency-sensitive, real-time device interactions.

#### 4.3.2. Based on Weighted Sum

Weighted sum-based trust aggregation techniques are widely used due to their computational simplicity. This section of the research is organized in the form of Table 5.

Weighted sum-based trust aggregation techniques, which assign weights to different trust features and sum them to obtain a final trust value, were one of the mainstream approaches in early SIoT trust management research, with representative work mainly appearing around 2020 (see Table 5). However, these methods are highly sensitive to weight, and reasonable allocation of weights among features is crucial to model performance. To alleviate this problem, several studies have introduced adaptive weighting mechanisms: Talbi et al. [79] derive weights from interest request frequencies, Wei et al. [83] iteratively update weight coefficients via a micro-step strategy based on the deviation between computed trust values and satisfaction levels, and Sagar et al. [84] adjust weights according to node historical behavior.

This study suggests that future SIoT trust-management models should dynamically optimize the weighting of heterogeneous trust features by incorporating contextual information and the characteristics of different trust attacks. Furthermore, integrating machine-learning methods to uncover latent correlations among features can improve the rationality of weight assignment, thereby enhancing model adaptability and robustness in complex SIoT environments.

#### 4.3.3. Based on Other Techniques

In addition, there are some studies on other trust aggregation techniques, including but not limited to fuzzy logic and blockchain.

Chen et al. [89] proposed an adaptive trust management protocol for social IoT systems. It uses honesty as a trust attribute to detect and handle BMA, BSA, and SPA attacks; cooperativeness and community interest to handle DA attacks; and records each node’s trust information to address WWA attacks. Binh et al. [90] proposed a trust assessment model called REK, which calculates trust values by integrating three trust metrics: reputation, experience, and knowledge, to effectively defend against BMA and BSA. Xia et al. [91] proposed a framework for context-aware trust inference. The framework calculates different trust elements using a kernel-based nonlinear multivariate gray prediction model, etc., and fuses them using the fuzzy logic approach. This model is able to handle the BMA. Amiri Zarandi et al. [92] proposed a blockchain-based SIoT trust model that can defend against BMA and BSA, DoS, and other trust attacks. Ouechtati et al. [93] proposed a fuzzy logic-based model to filter malicious nodes, which calculates the degree of social relationship, measures the strength of the relationship existing between the sender and the referral, and performs fuzzy logic to detect the presence of GMA and SPA. Narang et al. [94] proposed a hybrid trust management framework based on probabilistic neighborhood overlap, which can effectively defend against multiple trust attacks, including BMA, SA, OOA, and BSA.

Overall, these trust aggregation techniques further enrich the SIoT trust-modeling toolbox but entail clear trade-offs. Fuzzy-logic-based models can naturally capture vague and contextual trust information, yet rely on expert-defined rules and demand careful validation. Blockchain-based frameworks offer tamper-resistant, auditable trust records, but their consensus and ledger-maintenance costs often conflict with lightweight, real-time SIoT requirements. Thus, compared with weighted-sum and machine-learning approaches, these methods provide advantages in uncertainty handling and data security, while still facing limitations in scalability and deployment practicality.

### 4.4. Defense Strategies Against Trust Attacks and Their Classification

Among the examined literature, one part clearly indicates the defensive measures to deal with each type of trust attack, and the other only mentions the types of trust attacks that can be dealt with, as well as the overall defensive strategies. The overall summarized results are shown in Table 6.

Table 6 summarizes defense strategies against various trust attacks reported in the existing literature. These strategies can be broadly categorized into five types: feature-based, policy-based, trust measurement-based, trust prediction-based, and emerging technology-based (e.g., machine learning, blockchain), which together constitute the comprehensive defense strategy classification system presented in Table 7.

Based on Table 7, defense strategies against different trust attacks exhibit distinct characteristics. Feature-based defenses, which appear in all attack categories, are highly versatile but depend critically on the careful selection and combination of trust features. Policy-based defenses mitigate attacks by dynamically adjusting interaction rules or constraining malicious behaviors; they emphasize flexible behavior management and rapid reaction, but require a good understanding of the network environment and timely policy updates. Trust-metric-based defenses focus on computing and analyzing trust values to evaluate node reliability, and therefore rely on accurate trust models and efficient trust computation. Emerging-technology-based defenses leverage advanced techniques such as machine learning and blockchain: machine learning can automatically identify and classify attack-related patterns to improve the intelligence of defenses, while blockchain can enhance the transparency and tamper-resistance of trust records through decentralization. However, these techniques also introduce nontrivial costs and deployment constraints. Overall, each defense category has its own strengths and limitations, so practical SIoT deployments should combine multiple strategies and tailor them to the specific network environment and attack types to form a comprehensive defense plan.

## 5. Comparative Analysis of Trust Attack Simulation

### 5.1. Simulation Environment and Tools

The simulation environment used in this study is summarized in Table 8.

### 5.2. Simulation Experimental Design

**Figure 11:**
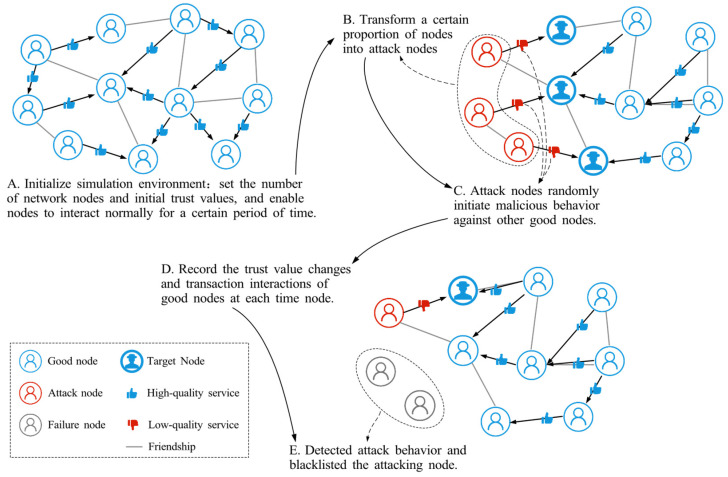
The experimental flow of trust attack simulation.

To make the impact of different trust attacks both observable and reproducible, the simulation is configured as a controlled data-generation process. During the attack phase, trust is updated only when malicious events occur and through the global time-decay mechanism. For each malicious event, the simulator logs the time step, interaction node ID, attack type, and the pre- and post-interaction trust values of both nodes. These raw logs are directly aggregated to compute the average trust of good nodes, and the transaction success rate. Because all data are generated by deterministic rules without exogenous noise, no additional data preprocessing is required. Figure 11 illustrates the steps of the trust attack simulation.

1. Initialize the simulation environment: as illustrated in step A, a synthetic SIoT interaction environment is instantiated with *N* nodes organized as an undirected social graph. Each node is randomly assigned *x* friend nodes to form its local social neighborhood, thereby approximating a sparse but connected friendship structure. The total length of the simulation is *S* time steps, the initial trust value of the nodes is *IT*, the number of allowed good nodes to interact with each other in a single time point is *TN*, and the condition for successful interaction is that the trust value of both interacting nodes is greater than the interaction threshold ϑ.
2. Implementing trust attacks: as shown in steps B and C, a certain percentage γ of nodes in the interaction process will be turned into attack nodes, and each attack node will launch an attack behavior with probability δ at each time step thereafter.
3. Record the node trust value: as shown in step D, during the simulation process, the trust value of each node *i* is denoted as $T_{i}$, and is dynamically updated after each time step. The update strategy comprises the following three approaches.
  - Trust increases through interaction: trust rises mainly in two cases: (i) a node (benign or malicious) provides high-quality service and receives positive feedback; (ii) nodes performing SPA or BSA submit self-promoting ratings. Both of these situations apply to Formula (1).
    (1) $$T_{i}\left(t+1\right)=T_{i}(t)+\alpha \times (1-T_{i}(t))$$
    Here, α represents the trust growth factor, $\left(1-T_{i}\right)$ characterizes the saturation effect: when $T_{i}$ is low, $\left(1-T_{i}\right)$ is large, meaning the same α yields a more pronounced trust increase. Conversely, when $T_{i}$ approaches 1, it becomes small, so a single positive event can only produce limited marginal gains. This naturally constrains the trust value within the [0, 1] range, reflecting the practical constraint that “high-trust nodes cannot continue to rise significantly due to a single act of good behavior.”
  - Trust decreases through interaction: when a node provides low-quality service or is penalized after an attack is detected, its trust score will decrease.
    (2) $$T_{i}\left(t+1\right)=T_{i}(t)-\beta \times T_{i}(t)$$
    In this formula, β represents the trust reduction factor. This equation embodies relative penalties: at the same β value, the higher the current trust level, the greater the reduction. Conversely, nodes with low trust levels will not experience significant drops in trust even if they exhibit malicious behavior again, aligning with real-world scenarios.
  - Trust decay over time: in real SIoT scenarios, if a node remains unobserved for an extended period, its historical evidence should gradually diminish in influence. To model this forgetting effect, a time decay mechanism is applied to all nodes at each step.
    (3) $$T_{i}\left(t+1\right)=T_{base}+(T_{i}(t)-T_{base})\times (1-\rho )$$
    Here, $T_{base}$ denotes the default neutral trust level of a node when it has not engaged in interactive behavior for an extended period. ρ represents the trust forgetting rate.
4. Trust attack detection mechanism: for each node *i*, we maintain a suspicion score $S_{i}$ that accumulates statistical evidence of malicious behaviour with exponential time decay. At each time step, the score is updated as
  (4) $$S_{i}\left(t+1\right)=\mu \times S_{i}(t)+\Delta S_{i}(0<\mu <1)$$
  (5) $$\Delta S_{i}=\kappa \times \chi_{i}^{2}(t)$$
  where the increment $\Delta S_{i}$ is proportional to a chi-square deviation $\chi_{i}^{2}(t)$ between the observed and expected rating behaviour of node *i*, Let $n_{i}(t)$ be the total number of ratings issued by node *i* up to time *t*, $o_{i}^{-}(t)$ the number of negative ratings, and $o_{i}^{+}(t)=n_{i}(t)-o_{i}^{-}(t)$ the number of non-negative ratings. Assuming a normal, approximately balanced rating pattern, the expected counts of negative and non-negative ratings are $e_{i}^{-}(t)=0.5\times n_{i}(t)$ and $e_{i}^{+}(t)=0.5\times n_{i}(t)$, and the chi-square statistic is computed as
  (6) $$\chi_{i}^{2}\left(t\right)=\frac{{\left(o_{i}^{-}(t)-e_{i}^{-}(t)\right)}^{2}}{e_{i}^{-}(t)}-\frac{{\left(o_{i}^{+}(t)-e_{i}^{+}(t)\right)}^{2}}{e_{i}^{+}(t)}$$
  In this way, a node that persistently issues disproportionately many unfair negative ratings obtains a large $\chi_{i}^{2}(t)$ and hence a rapidly growing suspicion score, whereas occasional negative feedback only produces small, transient increases that are gradually forgotten by the decay factor μ. A node is finally classified as malicious only when its suspicion score exceeds a preset threshold and its own trust value falls below the blacklist threshold.
5. Simulation comparison metrics: during the simulation, we record the average trust value (*GAT*) and transaction success rate (*TSR*) of good nodes. *GAT* measures how severely trust attacks damage honest nodes’ reputation, while *TSR* captures their impact on network functionality and operational efficiency. Both metrics are computed as in Equations (7) and (8).
  (7) $$GAT=\frac{1}{(1-\gamma )\times N}\sum_{i=1}^{(1-\gamma )\times N}{GT}_{i}$$
  (8) $$TSR=\frac{STN}{TN}$$
  where γ denotes the percentage of attacking nodes, *N* denotes the total number of nodes in the network, (1−γ)×N then denotes the number of good nodes in the network, and ${GT}_{i}$ denotes the trust value of the ith good node. In Equation (8), *STN* represents the number of successful transactions, and *TN* denotes the total number of interactions among nodes at a given time step.

### 5.3. Simulation Parameter Configuration

The simulation parameters are shown in Table 9.

The network size was fixed at (*N* = 100) and the time horizon at (*T* = 100) time steps, representing a small-scale SIoT interaction scenario while keeping the computational cost manageable. With the initial trust value IT=0.6 and the transaction threshold ϑ=0.6, the system starts from a high-trust regime where all interactions succeed, and the impact of attacks and time decay gradually drives the trust values towards the threshold. The time-decaying benchmark trust $T_{base}\;=\;0.5$ and the blacklisting threshold σ=0.5 are aligned with the midpoint of the trust scale, so that nodes whose trust persistently falls below this neutral level are regarded as high-risk. The proportion of attacking nodes γ is varied between 10% and 50% to cover light to severe attack scenarios. Each node is assigned x∈[5,10] “friend” nodes to approximate a sparse yet socially connected SIoT graph, and OOA attacks use an On/Off alternation cycle C=5. The decay coefficient of the suspicion score over time is μ=0.9, so that older evidence becomes gradually less influential. In parallel, the global trust-forgetting rate ρ=0.02 controls the speed at which trust values revert toward $T_{base}$, reflecting the fading of both positive and negative impressions during long periods without interaction.

### 5.4. Analysis of Simulation Results

**Figure 12:**
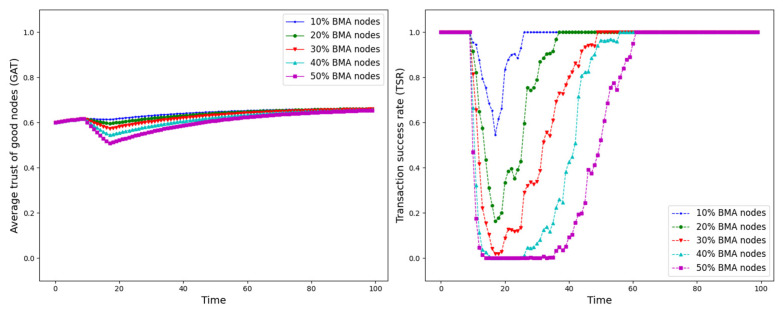
*GAT* and *TSR* with different proportions of BMA nodes.

**Figure 13:**
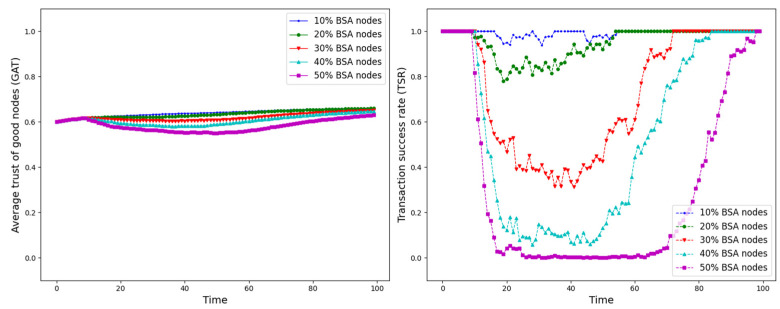
*GAT* and *TSR* with different proportions of BSA nodes.

**Figure 14:**
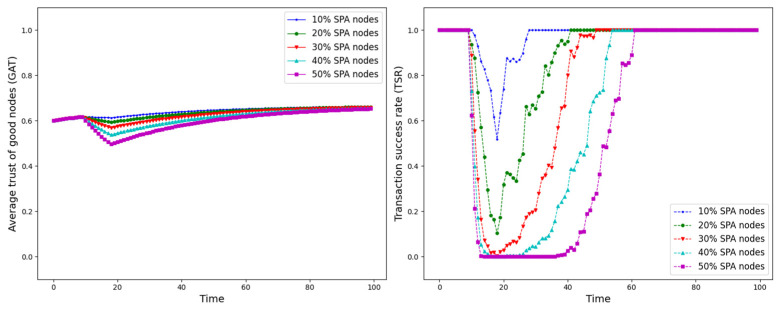
*GAT* and *TSR* with different proportions of SPA nodes.

**Figure 15:**
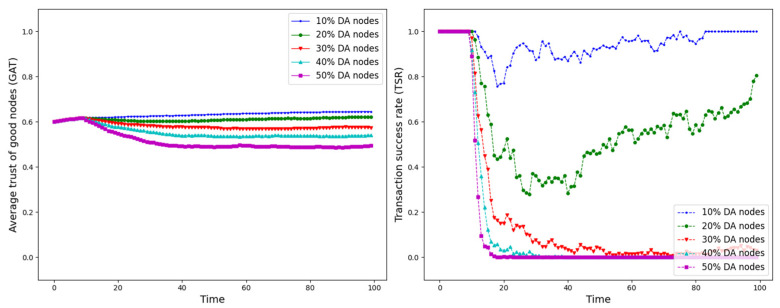
*GAT* and *TSR* with different proportions of DA nodes.

**Figure 16:**
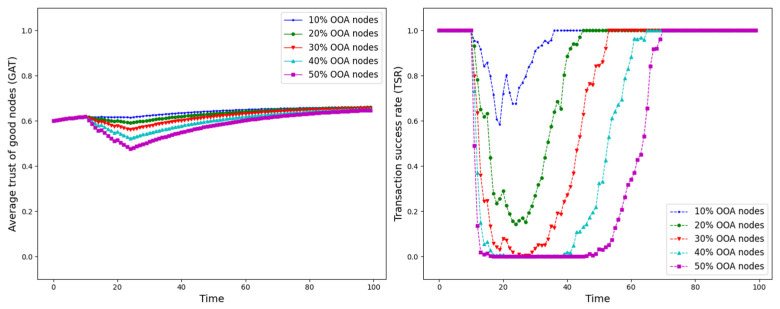
*GAT* and *TSR* with different proportions of OOA nodes.

**Figure 17:**
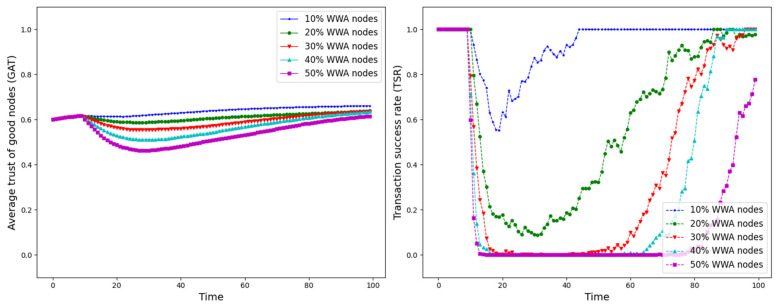
*GAT* and *TSR* with different proportions of WWA nodes.

**Figure 18:**
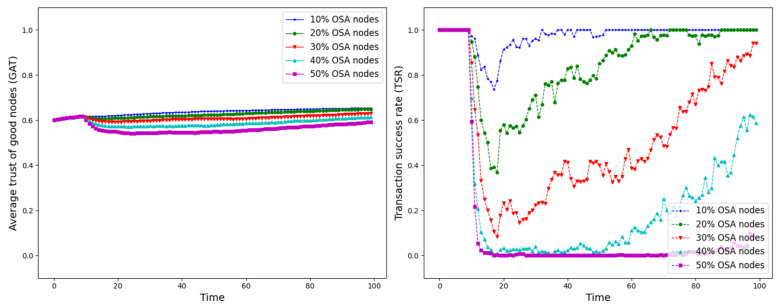
*GAT* and *TSR* with different proportions of OSA nodes.

The experiment is set up with all nodes interacting normally within the first 10 time points, and from the 10th time point onwards, different trust attacks are introduced to get the average trust of good nodes (*GAT*) and transaction success rate (*TSR*) situation during the whole interaction process. The results are shown in Figure 12, Figure 13, Figure 14, Figure 15, Figure 16, Figure 17 and Figure 18.

As shown in Figure 12, Figure 13, Figure 14, Figure 15, Figure 16, Figure 17 and Figure 18, the seven trust attacks exhibit consistent global patterns: the average trust of good nodes (*GAT*) experiences a brief decline at the onset of attacks and then gradually stabilizes or recovers as malicious nodes are detected and blacklisted, while the transaction success rate (*TSR*) generally forms a clear “V-shaped” or “U-shaped” collapse followed by recovery once the detection mechanism becomes effective. At the same time, as the proportion of malicious nodes increases from 10% to 50%, the trough of *TSR* often appears earlier and falls deeper, indicating that network service availability is more sensitive to attacks, while *GAT* shows stronger buffering and recovery capabilities in the global trend.

However, when focusing on local characteristics such as the slope of decline, the depth of the drop, the location of turning points, and the time needed for recovery, the two metrics show notable differences across attack types. From these local patterns, three key dimensions of attack capability can be extracted: disruption speed, attack strength, and stealthiness.

1. Disruption speed. Based on the slope at which *GAT* and *TSR* drop to their minimum values, the disruption speed can be ranked as: BMA, SPA and OSA fastest; OOA and WWA in the middle; and DA and BSA slowest. In the BMA, SPA and OSA scenarios, the *TSR* curves fall almost vertically once the attack starts, dropping to their minimum levels within roughly 20 time steps, while *GAT* also shows an early and clear decline. By contrast, OOA and WWA still drive *TSR* rapidly downward, but the turning points are noticeably delayed, leading to a more gradual degradation process. DA and BSA exhibit the slowest descent: the decreasing phase of *GAT* is longer, and *TSR* usually goes through a relatively mild downward or oscillating segment before reaching its minimum.
2. Attack strength. Using the maximum reduction of *GAT* and *TSR* as the criterion, the attack strength ranks as follows: BMA, SPA, OOA and WWA are the strongest; followed by DA; then OSA; and finally BSA. In BMA and SPA, *TSR* can be driven to very low levels in medium- and high-ratio scenarios, indicating a strong and abrupt destructive effect. OOA and WWA produce minima of similar depth but with longer persistence, so their overall impact is comparable to BMA and SPA. In contrast, DA typically yields slightly higher minima, suggesting a somewhat weaker destructive strength. OSA still causes substantial degradation but rarely collapses *TSR* completely, whereas BSA shows the smallest drop in *TSR*, allowing the network to retain a relatively higher transaction success rate even under 30% attack ratios.
3. Stealthiness. Considering the time required for GAT and TSR to recover from their minima, stealthiness can be ranked as: DA and OSA most stealthy; OOA, BSA and WWA at a medium level; and BMA, SPA and OOA least stealthy. In DA scenarios, TSR remains near zero for a long period after the collapse and only recovers slowly, forming an extended low platform that delays service restoration even after detection starts to work. OSA also shows long-lasting low levels and a slow upward trajectory, especially at medium and high ratios. In contrast, BMA, SPA and OOA rebound quickly after hitting bottom; their low-value segments are short and GAT stabilizes earlier, so malicious behavior is exposed more quickly and their temporal stealth is relatively weak.

### 5.5. Discussion

Building on the preceding review of existing studies and the analysis of simulation results, a comprehensive summary of seven trust attacks is presented in Table 10, including attack surface, attack capability, and recommended defense strategies.

Table 10 further illustrates that different trust attacks exhibit distinct attack surfaces and operational patterns. For BMA and BSA, the primary attack surface lies in manipulating social-layer recommendations and ratings. Strengthening rater credibility weighting, establishing abnormal rating-pattern detection, and restricting the propagation of suspicious feedback are essential to mitigating biased opinions and isolating collusive raters. SPA operates mainly through self-feedback channels; thus, limiting self-rating frequency, incorporating historical behavior auditing, and enforcing consistency checks between direct and indirect evidence can effectively reduce its impact. DA targets specific victims through differentiated service quality, making social-relationship constraints, structural comparison of community interactions, and long-term service disparity detection critical for identifying such selective unfairness. OOA exploits temporal patterns by alternating benign and malicious behaviors; therefore, time-sensitive trust decay, short-horizon behavioral consistency checks, and trust-cycle prediction can significantly improve resilience. WWA evades punishment by resetting identity, and can be mitigated through newcomer probation mechanisms, identity-association verification, and consistency analysis of behavioral trajectories. OSA relies on contextual cues to trigger opportunistic malicious actions; hence, context-aware trust updates, multimodal anomaly detection, and predictive early-warning models are effective countermeasures.

However, in real SIoT environments, attacks often appear in combined, dynamic, and context-dependent forms, meaning that single countermeasures remain insufficient, and a system-level, multi-source integrated defense framework is still required.

## 6. Challenges and Future Research

With the rapid development of SIoT, trust management models play a crucial role in ensuring system security and reliability. However, existing trust management models still have many limitations when facing increasingly sophisticated trust attacks. Future research needs to explore in depth the following aspects to cope with evolving trust attacks and emerging challenges.

- When constructing a trust management model, the selection of trust features directly affects the accuracy and efficiency of the model, and how to effectively select trust features is a key issue. Future research should construct trust feature sets in a more comprehensive and detailed way, including but not limited to node history behavior, interaction object reputation, quality of service, and so on. At the same time, feature combinations should be dynamically optimized for specific application scenarios and requirements by leveraging contextual information, so as to improve the adaptability and robustness of SIoT trust models [95].
- The topology of SIoT is heterogeneous and highly dynamic [96], and its services are also time-sensitive. Building trust management models with efficient scalability is therefore a major challenge. Future research should focus on developing heterogeneous scaling techniques that support rapid model expansion and flexible deployment for large-scale, high-complexity SIoT scenarios, while still ensuring accurate and timely trust assessment.
- The limited processing capabilities of SIoT devices and the heterogeneous and dynamic infrastructure expose multiple vulnerabilities [97], while trust management depends on large-scale user data, which exacerbates privacy risks. Future research must therefore carefully balance privacy protection and utility [98]. On the one hand, user data can be protected by encryption to prevent data from being stolen or tampered with during transmission and storage. On the other hand, privacy protection mechanisms, such as differential privacy [99] and federated learning [100], can be designed to enable effective trust assessment of user data while still protecting user privacy.
- Existing simulation tools for SIoT trust management have difficulty modeling large-scale dynamic device interactions, complex network environments, and realistic user behaviors, and context-based, efficient, and flexible simulation tools should be developed in the future to provide a reliable platform for research and testing [101]. In parallel, emerging technologies such as blockchain and machine learning provide new opportunities and challenges for SIoT trust management. Future research should explore their deeper integration with SIoT trust mechanisms and leverage their respective strengths to build safer, more efficient, and more intelligent trust management systems [102].

## 7. Conclusions

SIoT, as an emerging field, integrates the characteristics of social networks and IoT, which makes the interaction between devices, users, and services closer, but trust attacks become a serious challenge. In this paper, based on the in-depth study of SIoT architecture and trust management process, seven common trust attacks (BMA, BSA, SPA, DA, WWA, OOA, OSA) in SIoT are comprehensively analyzed. By organizing and summarizing the related literature in the last 10 years, the strategies of existing trust models in dealing with different types of trust attacks are comprehensively presented. In addition, trust-attack simulation and comparison experiments are designed and implemented to intuitively reveal how different trust attacks affect node trust and system transaction processing capability. Under the proposed trust-update and detection mechanisms, the results showed the attack capabilities of different trust attacks, confirming that different attack strategies shape both reputation evolution and service availability in distinct ways. Based on the above results of literature summarization and experimental analysis, many challenges faced by the existing trust management mechanisms in SIoT are summarized, including the selection of trust features, heterogeneity and extensibility of network topology, privacy security protection, simulation tool development, and convergence of emerging technologies, and a series of outlooks on the future research directions are proposed to address these challenges.

This work provides a systematic, up-to-date primer that enables readers to rapidly grasp core concepts for practical SIoT deployments. By elucidating the mechanisms and behavioral signatures of diverse trust attacks, it offers actionable guidance for designing trust management models capable of handling complex trust attacks. Nonetheless, the present simulations are intentionally designed as a conceptual and illustrative baseline: they employ simplified trust-update policies, fixed-size synthetic topologies, and do not yet capture realistic factors such as measurement noise, multi-hop trust propagation, node mobility, or coordinated adversaries. In future work, we will extend the framework with more comprehensive trust-management and evaluation mechanisms, integrate context-aware and multi-hop trust evolution into the attack scenarios, and validate the model on real SIoT datasets and traces, thereby delivering more realistic analyses of attack mechanisms and stronger empirical evidence to support defense strategy development.

## Figures and Tables

**Table 1:** Statistical analysis of existing review topics on SIoT.

Topics	[6]	[7]	[8]	[9]	[10]	[11]	[12]	[13]	[14]
Development of SIoT	✓			✓	✓				✓
Differences between SIoT and IoT					✓				
Latest activities in SIoT									✓
Foundational technologies in SIoT				✓					
Architecture of SIoT		✓	✓	✓	✓	✓	✓		
Social relationships			✓	✓		✓	✓		✓
Fundamental concepts and attributes of trust	✓	✓	✓	✓	✓	✓	✓		✓
Trust management process and components	✓	✓	✓	✓	✓	✓	✓	✓	✓
Trust management constraints		✓							
Classification of trust management models in existing research	✓	✓	✓	✓	✓	✓	✓	✓	✓
Trust attack threats in SIoT	✓	✓		✓	✓	✓	✓	✓	✓
Future research directions for SIoT	✓		✓				✓		✓
Challenges in SIoT			✓	✓	✓	✓			
Applications of SIoT			✓	✓			✓	✓	
Trust management platform					✓		✓		✓
Application platforms and tools of SIoT			✓			✓	✓		✓
Datasets of SIoT						✓			✓

“✓” indicates that the corresponding reference explicitly covers the topic.

**Table 2:** Comparison of the literature of the same type of review.

Ref	Year	SIoT-A	SR	T-C&A	TM-P	TA-N	TA-D	TA-S
[6]	2015	×	×	✓	✓	5	~	×
[7]	2016	✓	×	✓	✓	6	×	×
[8]	2019	✓	✓	✓	✓	6	×	×
[9]	2020	✓	✓	✓	✓	6	✓	×
[10]	2020	✓	×	✓	✓	7	×	×
[11]	2020	✓	✓	✓	✓	7	×	×
[12]	2022	✓	✓	✓	✓	7	✓	×
[13]	2023	×	×	×	✓	7	~	×
[14]	2024	×	✓	✓	✓	7	×	×
Our paper		✓	✓	✓	✓	7	✓	✓

Fully Covered: ✓, Not Covered: ×, Partially Covered: ~. SIoT-A: SIoT architecture, SR: Social Relationship, T-C&A: Concepts and Attributes of Trust, TM-P: Trust Management Process, TA-N: Number of described trust attacks, TA-D: Defense Against Trust Attacks, TA-S: Trust Attacks Simulation.

**Table 3:** Difference between the trust attack and the common cyberattacks.

Comparison Item	Cyber Attack	Trust Attack
Attack target	Disrupting system functions, stealing information, or blocking services, etc.	Manipulating trust mechanisms, misleading trust decisions, etc.
Attack mode	Exploit vulnerabilities, resource exhaustion, data theft, etc.	Forgery or falsification of trust information to interfere with trust assessment
Stealthiness	The stealthiness of different attacks varies widely	More covert
Scope of influence	Affect the system function, data security, cause business interruption, economic loss, etc.	Confusing the trust relationship between nodes, destroying the collaboration mechanism, etc.
Difficulty of detection	The difficulty of preventing different attacks varies greatly	Higher

**Table 4:** ML-based trust models.

Ref.	Year	Trust Features	ML Methods	Trust Attacks
[63]	2019	reputation, honesty, quality of provider, similarity, rating frequency, direct experience	MLP	BMA, BSA, SPA, DA
[64]	2019	reputation, honesty, quality of provider, similarity, direct experience, rating frequency, evaluation trend	MLP	BSA, BMA, SPA, DA
[65]	2020	goodness, usefulness, perseverance	iSVM	BSA, BMA, DA, WWA, OSA, OOA
[66]	2021	reputation, rating frequency, similarity, community of interest, honesty	MLP	BMA, BSA, SPA, DA, OSA
[67]	2021	direct trust, recommended trust, relative centrality	MLP	BMA, BSA, OSA
[68]	2021	recommendation, honesty, reputation, social similarity, knowledge	MLP, DBN	OSA, OOA, WWA
[69]	2023	direct Trust, reliability/benevolence, recommendation, degree of relationship	MLP	NA
[70]	2023	trust value, vote, vote similarity, users similarity, quality of provider, rating-frequency, rating-trend	Linear Regression	BMA, BSA, SPA, OOA, DA, OSA
[71]	2023	packet loss rate, delay, throughput	LSTM	BMA, BSA
[72]	2023	integrated degree of Influence, malicious transaction history, social sentiment score, ageing trust, feedback heterogeneity, feedback per service ratio	General	OOA, BSA
[52]	2025	centrality, social relationship factor, recommendation, computational and energy, capability, goodness, usefulness, perseverance, fluctuation	MLP	DA, OOA, WWA, OSA

**Table 5:** Weighted sum-based trust models.

Ref.	Year	Trust Composition	Trust Propagation	Trust Update	Trust Attacks
[73]	2016	computation power, context importance, confidence, feedback	Distributed	Hybrid	BMA, BSA, SPA, OOA
[74]	2017	direct trust, indirect trust, transaction factors	Distributed	Hybrid	OOA
[75]	2017	direct trust, Centrality, Cooperativeness, community interest, Service Score	Distributed	Time-driven	OOA
[76]	2017	direct Trust, centrality, cooperativeness, community interest, energy, service score	NA	Event-drive	OOA
[77]	2018	independent metrics, dependent metrics	Distributed	Event-drive	BMA, BSA
[78]	2019	intimacy, service feedback, sociability, transaction importance	Distributed	Event-drive	BMA, BSA, SPA, OOA, OSA
[79]	2019	direct trust, indirect trust	Distributed	Hybrid	BMA, SPA, DA, WWA
[80]	2020	honesty, cooperativeness, community of Interest, competence	Distributed	Time-driven	BMA, BSA, SPA, OSA
[81]	2020	competence, willingness	Distributed	Hybrid	BMA, BSA, SPA, DA, WWA, OSA
[82]	2020	context-based trust, reputation	Distributed	Hybrid	BMA, BSA, SPA, OSA
[83]	2020	direct interaction, direct interaction, direct relationship, indirect relationship	Hybrid	Hybrid	BMA, BSA, SPA, WWA
[84]	2020	similarity (community-of-interest, friendship, co-work), recommendation	Distributed	Hybrid	BMA, BSA
[85]	2020	service reliability, feedback validity, capability, activity, social relationship	Hybrid	Hybrid	BMA, SPA
[86]	2022	capability, commitment, satisfaction	Distributed	Time-driven	BMA, BSA, SPA, DA, OSA
[87]	2022	QoS, recommendation, trust uncertainty, context	Distributed	Hybrid	BMA, BSA, SPA, DA, WWA, OSA
[88]	2024	direct trust, recommendation, social similarity	Na	Event-drive	OOA

**Table 6:** Defense strategies of trust models against different trust attacks.

Ref.	Defense Strategies Against Trust Attacks
[52]	DA, OOA, WWA, OSA: centrality, social relationship factor, recommendation, computational and energy, capability, goodness, usefulness, perseverance, fluctuation
[63]	BMA, BSA, SPA, DA: trust feature aggregation and attack classification with MLP models
[64]	BMA, BSA: direct experience, rating frequency, QoS, honesty, reputation; SPA: similarity, QoS, honesty, reputation; DA: evaluation trends, QoS, reputation
[65]	BMA, BSA, DA, OOA, WWA, OSA: goodness score, usefulness score, perseverance score
[66]	BMA, BSA, SPA, DA, OSA: classifying trust attacks using ANN models
[67]	BMA: assign tasks based on transaction importance; BSA: weighting of referrer trust values; OSA: double the penalty for unsatisfactory events
[68]	OSA: honesty, reputation, cooperativeness, social similarity; OOA: reputation; WWA: cooperativeness, reputation
[70]	BMA, BSA, SPA, OOA, DA, OSA: trust value, vote, vote similarity, users similarity, quality of provider, rating-frequency, rating-trend
[71]	BMA, BSA: packet loss rate, delay, throughput
[72]	BSA, OOA: social sentiment score, ageing trust, feedback heterogeneity, feedback per service ratio
[73]	BMA: contextual feedback, multiple contextual factors; BSA: assessing feedback credibility; SPA: context; OOA: feedback variance, multiple contextual factors
[74]	OOA: secondary penalties, trust predictions
[75]	OOA: introduction of expected trust, trust cycle update
[76]	OOA: multi-attribute aggregation, dynamic update of trust, trust thresholds
[77]	BMA: recommendation trust; BSA: context
[78]	BMA, BSA: recommendation trust; SPA: service feedback; OOA, OSA: trust prediction
[79]	BMA: recommendation trust; SPA: indirect trust, relevance testing; DA: community of interest grouping; WWA: historical trust values, third-party recommendations
[80]	BMA: collaborative, communities of interest; BSA: honesty; SPA: historical behavior, recommendations; OSA: dynamically updated trust
[81]	BMA: multi-source trust dilution; BSA: threshold constraints; SPA: historical service records; DA, WWA: social relationship constraints; OSA: service penalty mechanisms
[82]	BMA, BSA: dynamically adjusting rater credibility; SPA: limiting own ratings; OSA: context
[83]	BMA, BSA: record integrity of blockchain; SPA: integrity and tamperability of blockchain; WWA: long-term behavioral records of blockchain
[84]	BMA: trust consistency testing; BSA: direct and indirect trust
[85]	BMA, SPA: normalization of trust
[86]	BMA, BSA: service feedback; SPA: historical experience; DA: social experience; OSA: dual penalty factor
[87]	BMA, BSA, SPA, DA, WWA, OSA: limiting own ratings, dynamically adjusting rater credibility, context
[88]	OOA: direct trust threshold
[89]	BMA, BSA, SPA: honesty; DA: cooperativeness, community-interest; WWA: historical trust, declining trust
[90]	BMA, BSA: reputation, experience, knowledge, time
[91]	BMA: service feedback
[92]	BMA, BSA: information entropy, limiting access to weak social connections, direct and indirect trust
[93]	BSA, SPA: social relationships
[94]	BMA: limit repeated attacks; BSA: multi-node evaluation prevents manipulation; OOA: evaluate feedback credibility

**Table 7:** Classification of defense strategies for trust attacks.

Trust Attacks	Category Classification	Refs.
BMA	Based on feature	[64,65,73,80,86,89,90,91]
	Based on policies	[67,81,82,84,85,87,88,92,94]
	Based on trust metrics	[77,78,79]
	Based on emerging technologies	machine learning: [63,66]; blockchain: [83]
BSA	Based on feature	[64,65,77,80,86,89,90,93]
	Based on policies	[67,73,81,82,87,88,94]
	Based on trust metrics	[78,84,92]
	Based on emerging technologies	machine learning: [63,66]; blockchain: [83]
SPA	Based on feature	[64,77,78,80,81,86,89,93]
	Based on trust metrics	[79]
	Based on policys	[67,82,87]
	Based on emerging technologies	machine learning: [63,66], blockchain: [83]
DA	Based on feature	[64,65,86,89]
	Based on policies	[79,81,87]
	Based on emerging technologies	machine learning: [63,66]
WWA	Based on feature	[65,68,89]
	Based on trust metrics	[79]
	Based on policies	[81,87]
	Based on emerging technologies	blockchain: [83]
OOA	Based on feature	[68,77]
	Based on trust prediction	[74,78]
	Based on policies	[73,75,76,94]
OSA	Based on feature	[65,68,82]
	Based on trust prediction	[78]
	Based on policies	[67,80,81,86,87]
	Based on emerging technologies	machine learning: [63,66]

**Table 8:** Simulation experiment environment.

Category	Params	Notes
Hardware	CPU	Intel(R) Xeon(R) Gold 5218, 2.30 GHz
	GPU	4×NVIDIA GeForce RTX 3090, 24 GB
	RAM	DDR4-2666 MHz ECC, 504 GB
Software	OS	Ubuntu 20.04.6 LTS
	Python	3.10.14
	Numpy	1.24.3
	Matplotlib	3.7.2

**Table 9:** Simulation parameter settings.

Params	Description	Value
*N*	Number of nodes in the network	100
*S*	Total simulation time	100
*IT*	Initial trust value of nodes	0.6
*TN*	Number of transaction interactions per round	1000
$T_{base}$	Time-decaying benchmark trust	0.5
ϑ	Trust value threshold for successful transaction interaction	0.6
γ	Percentage of attacking nodes	10~50%
σ	The trust threshold for blacklisting attacking nodes	0.5
ρ	Trust forgetting rate	0.02
μ	Suspicious score decay coefficient at each time step	0.9
*C*	On/Off attack alternation cycle	5
*x*	The number of “friend nodes” for each node	x∈[5,10]

**Table 10:** Comparative analysis of attack surfaces, disruption capabilities, and defense strategies for seven trust attacks in SIoT.

Attacks	Attack Surfaces	Attack Capability			Suggested Defense Strategies
		Disruption Speed	Attack Strength	Stealthiness	
BMA	Recommendation/rating manipulation	H	H	L	Recommendation trust; Rater credibility weighting
BSA	Recommendation/rating manipulation	L	L	M	Recommendation trust; Correlation analysis of rating patterns
SPA	Self-rating manipulation	H	H	L	Self-rating limitation; Trust consistency checks
DA	Targeted service interactions	L	M	H	Social relationship constraints; Long-term behavior profiling
OOA	Temporal pattern of service behaviour	M	H	L	Time-weighted trust decay; short-term consistency detection; Trust cycle prediction
WWA	Identity management and network admission	M	H	M	Trial period for new nodes; Identity association verification
OSA	Context-dependent service interactions	M	L	H	Context-aware trust updates; Trust prediction and early alerts;

H: high (strong/fast/highly stealthy in the corresponding dimension); M: medium; L: low.

## Data Availability

The data that support the findings of this study are available from the corresponding author upon reasonable request.
